# An Opportunistic Cooperative Packet Transmission Scheme in Wireless Multi-Hop Networks

**DOI:** 10.3390/s19214821

**Published:** 2019-11-05

**Authors:** Yating Gao, Guixia Kang, Jianming Cheng

**Affiliations:** 1 Key Laboratory of Universal Wireless Communications, Ministry of Education, Beijing University of Posts and Telecommunications, Beijing 100876, China; YTGao@bupt.edu.cn (Y.G.); chengjm@bupt.edu.cn (J.C.); 2 Wuxi BUPT Sensory Technology and Industry Institute CO.LTD, Wuxi 214000, China

**Keywords:** wireless multi-hop networks, cooperative routing, opportunistic routing, transmission beamforming, end-to-end transmission reliability and delay

## Abstract

Cooperative routing, combining cooperative communication in the physical layer and routing technology in the network layer, is one of the most widely used technologies for improving end-to-end transmission reliability and delay in the wireless multi-hop networks. However, the existing cooperative routing schemes are designed based on an optimal fixed-path routing so that the end-to-end performance is greatly restricted by the low spatial efficiency. To address this problem, in this paper an opportunistic cooperative packet transmission (OCPT) scheme is explored by combining cooperative communication and opportunistic routing. The proposed scheme divides the multi-hop route into multiple virtual multiple-input-multiple-output (MIMO) transmissions. Before each transmission, based on the idea of opportunistic routing, a cluster head (CH) is introduced to determine the multiple transmitters and multiple receivers to form a cluster. Then, the single-hop transmission distance is defined as the metric of forward progress to the destination. Each intra-cluster cooperative packet transmission is formulated as a transmit beamforming optimization problem, and an iterative optimal beamforming policy is proposed to solve the problem and maximize the single-hop transmission distance. CH organizes multiple transmitters to cooperatively transmit packets to multiple receivers with the optimized transmit beamforming vector. Finally, according to the transmission results, the cluster is updated and the new cooperative transmission is started. Iteratively, the transmission lasts until the destination has successfully received the packet. We comprehensively evaluate the OCPT scheme by comparing it with conventional routing schemes. The simulation results demonstrate that the proposed OCPT scheme is effective on shortening the end-to-end transmission delay, increasing the number of successful packet transmissions and improving the packet arrival ratio and transmission efficiency.

## 1. Introduction

In recent years, the rapid development of the Internet of Things (IoT) and 5G network has enabled wireless self-organizing multi-hop network wider applications in some of the harshest environments such as volcanoes, hurricane-affected regions, and underground mines. In such challenging environments, the issue of reliable and timely communication from end to end has received considerable critical attention because of the infrastructure-less property and the unstable nature of the wireless medium [1]. Considering that most IoT enabled nodes are equipped with a wireless transceiver to exchange data with other neighboring nodes, and, when necessary, to relay packets via neighboring nodes to destinations that are not within direct communications, the main challenge faced by many researchers is how to design an efficient routing scheme to improve the end-to-end performance of packet transmission such as transmission delay and packet delivery ratio [2,3]. It is now well established from a variety of studies [4,5], that the cooperative routing and opportunistic routing are proposed to increase the reliability and efficiency of end-to-end transmission by utilizing the broadcast advantage of wireless communications and creating diversity gains.

Cooperative routing is a cross layer routing scheme created by combining cooperative communication technology in the physical layer and routing technology in the network layer. It can effectively mitigate wireless fading and improve the reliability of wireless networks by allowing several single-antenna nodes to collaborate with each other and forward each node’s packet to the intended destination node [6]. The existing cooperative routing schemes [7,8,9,10,11,12,13,14,15] are implemented based on one or more optimized fixed end-to-end paths and can be classified into two categories. The first category is performed by finding the initial shortest path first and then improving the performance using cooperative techniques [9,10,11,12]. In these schemes, an initial non-cooperative end-to-end path is first established by the on-demand routing schemes [16,17,18] or energy-aware schemes [19], then the cooperative relays will be selected around the nodes on the initial path to participate in the cooperative transmission. This kind of cooperative routing scheme can improve the end-to-end performance to a certain extent compared with the conventional non-cooperative schemes. In fact, the effect of cooperation on the performance should be considered in searching the optimal end-to-end route. Therefore, the second category is designed by taking the effect of cooperation into the calculation of link cost, then the optimal end-to-end path is established based on the proposed link cost [13,14,15]. Such cooperative routing approaches, however, need to periodically evaluate the cooperative effect and update the link cost before selecting the optimal path. For this reason, the network-wide calculation of cooperative effect is not practical and can result in more overhead for the system. More importantly, the existing cooperative routing over a fixed optimal path cannot make the best of the spatial resource of the network, which largely limits the end-to-end transmission delay and reliability.

Opportunistic routing [20,21,22,23,24,25] has been proposed to support the transmission without a fixed path and overcome the unreliable wireless links by exploiting the broadcast nature of wireless transmission and path diversity in dense networks [26]. Instead of relying on one next-hop node to forward a data packet in the optimal path based routing, opportunistic routing pre-determines a set of candidate relays with a priority order based on the instantaneous channel conditions and allows them to overhear the current packet transmission. The highest-priority relay that has successfully received the packet will be selected as the next actual forwarder. By dynamically selecting the forwarder from a set of multiple candidate receivers, opportunistic routing can significantly reduce the number of packet retransmissions caused by link failures. Most existing opportunistic routing schemes propose candidate selection and prioritization based on some heuristics, like the geographic random forwarding (GeRaF) [21], the extreme opportunistic routing (ExOR) [22], the geographic collaborative forwarding (GCF) [23], the quality of service aware geographic opportunistic (EQGOR) [24] and the energy efficient opportunistic routing (EEOR) [25]. These opportunistic routing schemes can greatly improve routing delay and reliability with unreliable links, but in general fails to provide optimality. Moreover, the packet transmission in these opportunistic routing schemes is equivalent to an iterative broadcast process, and only the best node is selected as the forwarder. In this case, most nodes that have received the packet cannot participate in forwarding packets, which lowers the end-to-end transmission efficiency and still remains to be improved.

Motivated by the above shortcomings, in this paper, we propose an opportunistic cooperative packet transmission (OCPT) scheme to further improve the end-to-end transmission performance. In contrast with the existing schemes, the importance and originality of this study are that it divides the multi-hop route into multiple virtual multiple-input-multiple-output (MIMO) transmissions by combining the cooperative transmission and opportunistic routing. Before each transmission, based on the idea of opportunistic routing, a cluster head (CH) is introduced to determine the multiple transmitters and multiple receivers from its neighbor nodes so that a cluster can be formed. Then, the intra-cluster cooperative transmission is formulated as a transmit beamforming optimization problem, and an iterative optimal beamforming policy is proposed to obtain the optimal transmit beamforming vector and maximize the single-hop transmission distance. CH organizes the intra-cluster cooperative packet transmission, where multiple transmitters cooperatively transmit the packet to multiple receivers simultaneously based on the optimized transmit beamforming vector. Finally, according to the transmission results, the CH node is updated and the new cooperative transmission is started. Iteratively, the transmission lasts until the destination has successfully received the packet. Designed by opportunistic routing, the proposed OCPT scheme can reduce the unnecessary retransmission resulting from the packet loss and enhance the high reliability of end-to-end transmission. Meanwhile, the optimized intra-cluster cooperative communication can maximize the single-hop transmission distance and shorten the end-to-end transmission delay.

The remainder of this paper is organized as follows. Section 2 reviews the related work. Section 3 illustrates the system model. Section 4 presents the proposed OCPT scheme. The performance evaluation results of OCPT scheme are provided in Section 5. Finally, the conclusion is drawn in Section 6.

Notation: We use boldface to denote vectors. ${\left(\cdot \right)}^{T}$ denotes transpose, while ${\left(\cdot \right)}^{†}$ denotes Hermitian (conjugate) transpose operators. The norm of a vector x is defined as $∥x∥=\sqrt{x^{†}x}$. $E\left\{\cdot \right\}$ denotes expectation operations.

## 2. Related Work

Since the timely and reliable end-to-end data packet delivery is very important for an intelligent surveillance system and security alarm system, a number of previous routing protocols [9,10,11,12,13,14,15,16,17,18,19,27] have been proposed to improve the end-to-end transmission performance. Dynamic source routing (DSR) [17] and ad hoc on-demand distance vector (AODV) [16] are two typically well-known routing protocols belonging to ad hoc routing. AODV is formulated as a loop-free, on-demand, single path, distance vector protocol, where a broadcast route discovery algorithm and the unicast route reply message were proposed to find a best path. As the extensions of on-demand routing, several routing protocols [18,19,27] were proposed based on various criteria and design issues. For example, AODV-ETX [18] defined the link cost as the expected transmission times (ETX) to find an optimal routing path with the minimum total ETX, the minimum total energy routing (MTE) [19] was designed to find the minimum-energy route in network, and split multipath routing (SMR) [27] took advantage of two on-demand maximally disjoint routes for each session to improve the transmission reliability.

Furthermore, cooperative communication models [9] were introduced as a new element in designing the routing protocols for wireless ad hoc networks due to the fact that many-to-many cooperative communication can improve the transmission reliability via transmit diversity compared with the classical point-to-point transmission. Khandani et al. [10] introduced the cooperative communication at the physical layer into the multi-hop routing and proposed the cooperation along the best non-cooperative path (CAN). In CAN, the last few nodes along the selected non-cooperative path are employed as the relay nodes. M. Elhawary et al. [11] designed a cluster-based routing (CwR) scheme, where an on-demand initial path is firstly established and then each node in the path starts the cluster recruitment to establish a cluster with itself as the cluster head (CH). The cluster-to-cluster packet transmission was designed to beamform the packet to each receiver so that the transmission reliability can be improved. On the basis of CwR, a cluster-based cooperative packet transmission (CCPT) [12] scheme was further proposed to improve the energy efficiency and reduce the end-to-end transmission delay. In contrast with CwR, the design of inter-cluster cooperative transmission focused on the successful reception of the receiving CH rather than the whole receiving cluster, which can enhance the robustness of transmission on the best path and effectively reduce the transmission times, so that the end-to-end transmission delay can be shortened.

We adopt AODV-EXT and CCPT as the baselines. It can be seen that AODV-ETX is a special case of CCPT with the predefined maximum size of each cluster $N_{P}=1$. Compared with AODV-ETX, CCPT can considerably enhance the reliability of single-hop packet transmission on the initial path by designing the cluster-based cooperative transmission, which can create the transmit diversity. However, on the one hand, the construction of a fixed initial path may introduce the excessive delay and signalling overhead. On the other hand, the transmission on a fixed initial path cannot adopt to the frequent changes in a wireless network, which will result in much retransmissions, thereby increasing the end-to-end transmission delay.

Different from the above optimal path based routing schemes, the transmission of opportunistic routing is performed without a fixed route. S. Biswas et al. designed the classical opportunistic routing scheme ExOR [22], which shaped the main principles and building blocks of opportunistic routing. Generally, the basic operations of opportunistic routing [20,21,22,23,24,25,28,29,30,31,32,33,34] include three main steps: First, the source node broadcasts the data packet to a set of candidate relays instead of a single predetermined relay. All the candidate relays are prioritized according to a specific metric, for example, the geographical distance [21,22,23,24], the hop count [29,30], energy cost [25,28], ETX [31], and etc. Meanwhile, most of the existing schemes consider the optimization of the candidate relays set, i.e., removing some candidates from the candidate relays set especially in large wireless networks. In addition to the above-mentioned metric, the existing considerations also include connectivity [31,32], node contribution [33,34], etc. Second, when one relay node successfully receives the packet, it will become one of the candidate relays and broadcast an acknowledge (ACK) message. Third, a relay node acts as the actual forwarder and carries on the packet transmission only if no relay node with higher priority has explicitly acknowledged receipt of it. This process continues until the destination has successfully received the packet.

Overall, opportunistic routing can take advantage of the spatial resource by using the broadcast nature of radio communication and opportunistically picking the best candidate relay to forward the packet, which can guarantee the packet progresses towards the destination at each hop and improve the end-to-end transmission reliability. However, only one node with the highest priority is selected as the actual forwarder, and other nodes that have successfully received the packet cannot participate in forwarding packets, which greatly limits the end-to-end transmission efficiency.

## 3. System Model

We consider a stationary wireless network (like an intelligent surveillance system and security alarm system) consisting of *N* nodes arbitrarily distributed in a two-dimensional area, where each sensor node stays stationary and is equipped with a single omnidirectional antenna. These nodes can self-organize to form a multi-hop network. Define the whole network as a connected undirected graph $G=\left(V,P\right)$, in which V is the set of nodes and $\left|V\right|=N$ is the number of nodes. P denotes the set of all the bi-directional wireless communication links between pairs of nodes. In the case where a node i∈V transmits a data packet *X* with the maximum power $P_{t}$, and another node j∈V has the chance to successfully decode *X* without the aid of any other nodes, we say a link $P_{i,j}\in P$ exits. The signal received at node *j* from node *i* can be expressed as

(1) $$y_{j}=h_{i,j}w_{i}X+z_{j},$$

where $z_{j}$ is a zero-mean, wide-sense stationary additive noise at node *j* with variance $\sigma_{j}^{2}$, and it is assumed to be independent of *X* and $h_{i,j}$. *X* is the unit of data packet with a fixed length of *L* information bits and satisfies $E[|X{|}^{2}]=1$. $w_{i}$ is the power control coefficient such that $|w_{i}{|}^{2}=P_{i}\leq P_{t}$. $h_{i,j}$ denotes the complex channel gain between node *i* and *j*. We assume that the channel between any two nodes is Rayleigh block fading, and $E[{\left|h_{i,j}\right|}^{2}]$ is inversely proportional to $d_{i,j}^{\alpha }$ [35], where $d_{i,j}$ denotes the distance between node *i* and *j*, and α is the path loss exponent. The received signal to noise ratio (SNR) at node *j* is given by

(2) $$\gamma_{j}=\frac{{\left|h_{i,j}w_{i}\right|}^{2}}{\sigma^{2}}=\frac{P_{i}{\left|h_{i,j}\right|}^{2}}{\sigma^{2}}.$$

Assume that error detection for packet loss is perfect; that is, a packet is correctly received only if all bits in this packet are decoded correctly. If a packet is not received correctly, this packet will be retransmitted by the corresponding sender. We assume that the packets are transmitted with uncoded *Q*-ary quadrature amplitude modulation (QAM) signalling, where *Q* is the constellation size for each signal symbol and can be any integer equal to or larger than 4. Each bit will experience independent Rayleigh fading, and the bit error rate (BER) at node *j*, denoted by $p_{i,j}^{b}\left(\gamma_{j}\right)$, can be approximated by [36]:

(3) $$p_{i,j}^{b}\left(\gamma_{j}\right)\approx c\exp\left(-\beta \gamma_{j}\right).$$

where *c* is a constant c=0.2, and $\beta =1.5/\left(Q-1\right)$. Then, the packet loss probability $p_{i,j}$ can be expressed as [37]

(4) $$p_{i,j}=1-{\left[1-p_{i,j}^{b}\left(\gamma_{j}\right)\right]}^{L}.$$

where *L* is the packet length in bits. If node *i* can reach *j*, i.e. $P_{i,j}\in P$, then by definition there must be $p_{i,j}<1$. According to a threshold-based approximate expression [14,38] of (4): $p_{i,j}\approx \min\left\{cL\exp\left(-\beta \gamma \right),1\right\}$, the neighbor set of node *i*, $N\left(i\right)$, consists of all reachable nodes of *i*:

(5) $$N\left(i\right)=\left\{j\in V\left|\frac{P_{t}{\left|h_{i,j}\right|}^{2}}{\sigma^{2}}\geq \gamma_{\mathrm{th}}\right.\right\},$$

where $\gamma_{th}=\log\left(cL\right)/\beta$.

Similar to in [21,24], we assume that nodes always have a means to acquire the geographical location information of their neighbors and where the destination is. Moreover, similar to [4,8], we assume that, within the range of N(i),∀i∈V, channel state information (CSI) of $P_{i,j},\forall j\in N\left(i\right)$ is available to node *i* and the signaling messages can be transmitted reliably with the negligible cost due to the fact that the length in bits of these messages is very small compared to the traffic data.

Furthermore, let $T_{i,j}$ represent the number of transmission attempts required for node *i*, with which, node *j* can achieve a successful packet reception. Considering that only finite end-to-end transmission delay can be afforded in practice, the maximum number of automatic repeat request (ARQ) retransmissions has to be bounded [39]. We set the maximum retransmission times as $N_{t}^{\max}$, then the expected transmission times (ETX) under the packet loss probability $p_{i,j}$ can be obtained by Equation (6).

(6) $$\begin{array}{cc} E\left[T_{i,j}\right] & =\sum_{k=1}^{N_{t}^{\max}}\left[k\cdot \left(1-p_{i,j}\right)\cdot p_{i,j}^{k-1}\right]+N_{t}^{\max}p_{i,j}^{N_{t}^{\max}}\\ & =\frac{1-p_{i,j}^{N_{t}^{\max}}}{1-p_{i,j}}, \end{array}$$

when $N_{t}^{\max}\to \infty$, $E\left[T_{i,j}\right]=\frac{1}{1-p_{i,j}}$. For each single-hop link $P_{i,j}\in P$, once node *j* fails in receiving the packet after $N_{t}^{\max}$ retransmissions, the packet will be dropped, and declared as a failed packet delivery.

It is assumed that all the nodes can always hold time synchronization, and each packet transmission takes one time slot. Given a source node $V_{s}$ and destination $V_{d}$, $V_{s}$ initiates a communication session to $V_{d}$. Similar to [10,40], we assume that each time there is only one active communication session, which is the one from $V_{s}$ to $V_{d}$. The end-to-end delay can be measured with the total transmission times, i.e., the total time slots consumed during the session.

## 4. Opportunistic Cooperative Packet Transmission

In this section, we propose an OCPT scheme that combines the cooperative transmission and opportunistic routing to improve the end-to-end transmission performance. We firstly design a cluster-based opportunistic cooperative routing scheme, in which CH determines transmitters and receivers from its neighbor set to form a cluster. Then, the intra-cluster cooperative transmission is formulated as a transmit beamforming optimization problem. An optimal beamforming policy is proposed to obtain the optimal transmit beamforming vector and maximize the single-hop transmission distance.

### 4.1. Cluster-Based Opportunistic Cooperative Routing

The proposed scheme starts with the packet broadcast of source node $V_{s}$. Before the data transmission, $V_{s}$ will broadcast a request-to-send (RTS) message where the node ID and the position of $V_{d}$ are included. Any neighbor node $i\in N\left(V_{s}\right)$ whose position to $V_{d}$ is closer than $V_{s}$ will send back a clear-to-send (CTS) message with its node ID to $V_{s}$ and be ready to overhear the upcoming data packet. Then $V_{s}$ will broadcast the data packet to the waiting receivers. If any receiver successfully receives the packet, it will broadcast an ACK message in the range of $N\left(V_{s}\right)$ composed of its node ID and position information to $V_{d}$.

We denote $S_{n}$ as the set of nodes that have successfully received the packet after the $\left(n-1\right)$ transmissions. $S_{1}=V_{s}$, $\left(S_{n+1}-S_{n}\right)$ represents the nodes that have successfully received the packet in the nth transmission. Considering that once a node is out of neighbor set $N\left(V_{d}\right)$ of the destination node, the channel between them is too weak for the obtention of CSI. In this case, the channel condition of each node to the destination is measured with the Euclidean distance of these two nodes. We suppose that all the nodes are prioritized by distance to the destination: The shorter the distance is, the higher the priority will be. The nth transmission is organized by a CH node $V_{n}$, which is selected from $\left(S_{n}-S_{n-1}\right)\cup V_{n-1}$ by priority. Thus, $V_{n}$ is the closest node in $S_{n}$ to the destination, i.e.,

(7) $$V_{n}=\underset{V_{n}\in \left(S_{n}-S_{n-1}\right)\cup V_{n-1}}{\arg\min}\left\{d_{V_{n},V_{d}}\right\},\forall n\geq 2.$$

Clearly, $V_{1}=V_{s}$. By resolving the ACK messages from the wireless medium, any node in $\left(S_{n}-S_{n-1}\right)\cup V_{n-1}$ will act as the CH $V_{n}$ only if no node with higher priority has explicitly acknowledged receipt of it, which can be achieved in a distributed fashion. Each transmission is based on a cluster $C_{n}$ with $V_{n}$ as the CH. The cluster members include all the cooperative transmitters and receivers, which are selected from the neighbor set of CH. Therefore, the main work of CH herein includes the following two parts:

- Perform the RTS/CTS message exchange to determine the cooperative transmitters and receivers.
- Organize the cooperative transmitters (including CH itself) to cooperatively transmit data packets.

Denote $T_{n}$ and $R_{n}$ as the transmitter and receiver set for the *n*th packet transmission, respectively. Any node $i\in N\left(V_{n}\right)$ has successfully received the packet can join in the follow-up cooperative transmission, therefore, the set of cooperative transmitters is obtained by

(8) $$T_{n}=S_{n}\cap N\left(V_{n}\right).$$

Thus, $T_{1}=\left\{V_{s}\right\}$. The packet broadcast process of the source node can be seen as the transmission without cooperation. Then, according to the broadcast nature of wireless mediums, any node $i\in N\left(V_{n}\right)$, which fails in receiving the packet and has the better channel condition than $V_{n}$ should act as the receiver, i.e., the set of receivers can be obtained as

(9) $$R_{n}=\left\{i\in N\left(V_{n}\right)|d_{i,V_{d}}\leq d_{V_{n},V_{d}}\right\}.$$

Both the transmitters $T_{n}$ and receivers $R_{n}$ form a cluster set $C_{n}=T_{n}\cup R_{n}$, which is a subset of neighbor set, i.e., $C_{n}\subseteq N\left(V_{n}\right)$. Similar to the broadcast of $V_{s}$, before the data transmission, CH $V_{n}$ will broadcast a RTS message, and any node $i\in N\left(V_{n}\right)$ satisfying (8) or (9) will reply with a CTS message to join $T_{n}$ or $R_{n}$. Once the transmitters and receivers are determined, $V_{n}$ will organize the intra-cluster cooperative packet transmission, i.e., the nodes in $T_{n}$ cooperatively beamform the packet to the nodes in $R_{n}$. As assumed in the Section 3, the control signaling messages in the range of $C_{n}$ (including RTS/CTS, obtention and exchange of CSI, required signaling messages for cooperative beamforming, and ACK messages) can be transmitted reliably with the negligible cost because of the short bit length.

**Figure 1:**
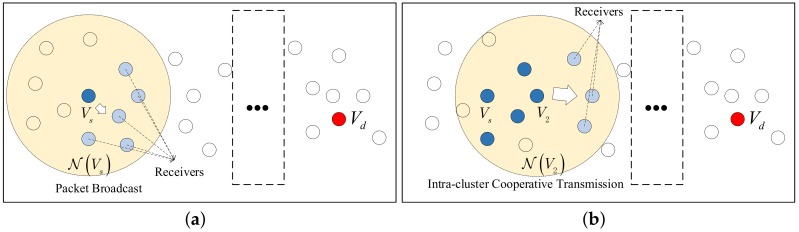
The cluster-based opportunistic cooperative routing. (**a**) Broadcast of source node $V_{s}$ (**b**) Intra-cluster cooperative transmission.

Figure 1 shows the proposed cluster-based opportunistic cooperative routing scheme. In Figure 1a, the cluster $C_{1}\subseteq N\left(V_{s}\right)$ is formed by the source node $V_{s}$ (blue node) and receivers $R_{1}$ (grey nodes), which are selected by (9). $V_{s}$ broadcasts the packet to $R_{1}$. Figure 1b shows the next-hop packet transmission after the packet broadcast. CH $V_{2}$ is selected from $S_{2}$ using (7), and the cluster $C_{2}\subseteq N\left(V_{2}\right)$ is formed by the cooperative transmitters $T_{2}$ (blue nodes) and receivers $R_{2}$ (grey nodes), which are determined by (8) and (9), respectively. Then, an intra-cluster cooperative packet transmission will be organized by $V_{2}$. The intra-cluster cooperative transmission including multiple transmitters $T_{2}$ and multiple receivers $R_{2}$ is equivalent to a virtual MIMO transmission, which fundamentally consists of multiple MISO links due to the fact that multiple receivers are separated from each other and difficult to cooperatively receive the data signal. The exact formulation of virtual MIMO transmission will be unfolded in Section 4.2.

It can be seen from Figure 1 that the multi-hop route is divided into iterative intra-cluster packet transmissions, each of which is performed as a virtual MIMO transmission among the cluster members. The formal description of our proposed opportunistic cooperative routing scheme is presented in Algorithm 1. Starting from source $V_{s}$, iteratively, the packet is cooperatively forwarded to the destination $V_{d}$. Unlike the conventional cooperative schemes, where a fixed routing path needs to be established first, the routing decisions of the proposed scheme, in contrast, are made in an online manner by choosing the transmitters and receivers for the upcoming transmission based on the actual transmission outcomes. Meanwhile, different from the conventional opportunistic routing, each transmission is performed as an intra-cluster cooperative communication instead of the broadcast of a single relay, so that the transmit diversity can be used to improve the reliability.

**Algorithm 1** Proposed opportunistic cooperative routing scheme
1:Initiate $S_{0}=\left\{V_{s}\right\},V_{1}=V_{s},T=S_{0}$, get R by (9).2:n←0. The source $V_{s}$ broadcasts the packet *X*.3:**while**$V_{d}$ fails in receiving *X* **do**4: n←n+1;5: Update $S_{n}$ as the set of nodes that have received *X*;6: Update $V_{n},T_{n}$ and $R_{n}$ by (7),(8) and (9);7: $V_{n}$ organizes the nodes in $T_{n}$ to transmit *X* to $R_{n}$ with cooperative beamforming.8:
**end while**



We introduce the intra-cluster cooperative communication in the next subsection. For simplicity of notation, drop the index $\left\{n\right\}$ of symbols in the following description.

### 4.2. Intra-Cluster Cooperative Communication

Set $N_{t}=\left|T\right|$ and $N_{r}=\left|R\right|$ as the number of transmitters and receivers, respectively. Let $w={\left[w_{1},w_{2},\cdots ,w_{n_{t}}\right]}^{T}\in C^{n_{t}}$ denote the transmit beamforming vector and $h_{j}={\left[h_{1,j},h_{2,j},\cdots ,h_{n_{t},j}\right]}^{T}\in C^{n_{t}}$ denote the complex channel coefficient vector between transmitters T and node j,∀j∈R. Under the virtual MIMO transmission model, the beamforming vector is used by T to transmit a zero-mean, unit-variance, common information signal *X* to all $N_{r}$ receivers. The corresponding received signal at node *j* can be expressed as

(10) $$y_{j}=h_{j}^{†}wX+z_{j},\forall j\in R.$$

Further, the received SNR at node *j* is then given by

(11) $$\gamma_{j}=\frac{{\left|h_{j}^{†}w\right|}^{2}}{\sigma_{j}^{2}}=w^{†}R_{j}w,$$

where $R_{j}\overset{\Delta }{=}\frac{h_{j}h_{j}^{†}}{\sigma_{j}^{2}}⪰0$.

Define the *single-hop transmission distance* ξ as the metric of forward progress towards the destination, i.e., $\xi_{i}=d_{V_{n},d}-d_{i,d}$, for i∈R. Considering that the end-to-end transmission delay is measured with the total transmission times, to shorten the total end-to-end transmission delay, the intra-cluster cooperative transmission should be designed to maximize the transmission speed, i.e., the forward progress of each single-hop transmission. Therefore, the intra-cluster cooperative transmission aims at maximizing the single-hop transmission distance.

To model the distribution of discrete variable ξ, without loss of generality, we suppose that $\xi_{1}<\xi_{2}<,\cdots ,<\xi_{N_{r}}$. $\xi =\xi_{i},i\in \left[1,\cdots ,N_{r}\right]$ only when receiver *i* has successfully received the packet and every receiver $j\left(j>i\right)$ has failed in receiving. Therefore, the expectation of single-hop transmission distance can be expressed as

(12) $$\begin{array}{cc} E\left[\xi \right] & =\sum_{i=1}^{N_{r}}\left\{\left(1-p_{T,i}\right)\cdot \prod_{j=i+1}^{N_{r}}p_{T,j}\cdot \xi_{i}\right\}\\ & =\sum_{i=1}^{N_{r}}\left\{\xi_{i}\cdot \left(\prod_{j=i+1}^{N_{r}}p_{T,j}-\prod_{j=i}^{N_{r}}p_{T,j}\right)\right\}, \end{array}$$

where $p_{T,j}$ is the function of w. Therefore, let $f\left(w\right)\overset{\Delta }{=}E\left[\xi \right]$, $g_{i}\left(w\right)\overset{\Delta }{=}\prod_{j=i}^{N_{r}}p_{T,j}$. Substituting (3), (4) and (11), $g_{i}\left(w\right)$ can be rewritten as the function of w,

(13) $$g_{i}\left(w\right)=\prod_{j=i}^{N_{r}}\left\{1-{\left[1-c\exp\left(-\beta \cdot w^{†}R_{j}w\right)\right]}^{L}\right\}.$$

Since $g\left(w\right)$ is a continuously differentiable function, the gradient of $g\left(w\right)$ can be given as

(14) $$\nabla_{w}g_{i}\left(w\right)=\sum_{j=i}^{N_{r}}\left\{-2cL\beta {\left[1-c\exp\left(-\beta \cdot w^{†}R_{j}w\right)\right]}^{L-1}\cdot \exp\left(-\beta \cdot w^{†}R_{j}w\right)\cdot R_{j}w\cdot \prod_{k\in \left[i,N_{r}\right]}^{k\neq j}p_{T,k}\right\}.$$

Furthermore, the gradient of $f\left(w\right)$ can be given.

(15) $$\nabla_{w}f\left(w\right)=\sum_{i=1}^{N_{r}}\left\{\xi_{i}\cdot \left[\nabla_{w}g_{i+1}\left(w\right)-\nabla_{w}g_{i}\left(w\right)\right]\right\}.$$

Under the total power constraint, maximizing the single-hop transmission distance is equivalent to find a transmit beamforming vector w that maximizes the expectation of single-hop transmission distance over a feasible set $\mathrm{Tr}\left(ww^{†}\right)\leq P_{t}$. Since $f\left(w\right)$ is an increasing function of the transmit power, the power constraint can be replaced with an equality constraint:

(16) $$\mathrm{Tr}\left(ww^{†}\right)=P_{t}.$$

Thus, the optimization problem can cast as

(17) $$\prod_{1}:\;w^{*}=\underset{w}{\mathrm{argmax}}\left\{f\left(w\right)\right\}$$

subject to the constraint (16). To compute the Karush–Kuhn–Tucker (KKT) conditions, the Lagrangian is

(18) $$L\left(w,\mu \right)=-f\left(w\right)-\mu \left[P_{t}-\mathrm{Tr}\left(ww^{†}\right)\right],$$

where μ is the Lagrangian multiplier for the constraint. Further, the first order condition is

(19) $$\nabla_{w}L\left(w,\mu \right)=-\nabla_{w}f\left(w\right)+\tilde{\mu }w,$$

where $\tilde{\mu }=2\mu$. Therefore, the solution to (19) must satisfy the following fixed point equation

(20) $$w_{FP}=\frac{1}{\tilde{\mu }}\nabla_{w}f\left(w_{FP}\right),$$

for some constant $\tilde{\mu }\in R$. It is difficult to give a closed-form solution based on (20), therefore, we consider to obtain a numerical solution for the maximization problem by a proximal gradient method [41].

Consider a strongly concave approximation [42] of $f\left(w\right)$ at $w=w_{n}$

(21) $$f\left(w\right)\approx f\left(w_{n}\right)+{\left(\nabla_{w}f\left(w_{n}\right)\right)}^{†}\left(w-w_{n}\right)-\frac{{∥w-w_{n}∥}^{2}}{2\lambda }:=u\left(w,w_{n}\right)$$

where λ is a positive constant. The first two terms in $u\left(w,\;w_{n}\right)$ are the first order Taylor series approximation of $f\left(w\right)$ at $w=w_{n}$. The last term is a proximal regularization term that is included to make $u\left(w,\;w_{n}\right)$ strongly concave. Instead of solving $\prod_{1}$, suppose that we iteratively solve $\prod_{2}$ to obtain $w_{n+1}$ from $w_{n}$.

(22) $$\prod_{2}:\;w_{n+1}=\underset{w}{\mathrm{argmax}}\left\{u\left(w,w_{n}\right)\right\},$$

subject to the constraint (16). It can be observed that the objective function $u\left(w,\;w_{n}\right)$ is a quadratic function with respect to $\left(w-w_{n}\right)$. Therefore, the solution of $\prod_{2}$ can be obtained in closed form as

(23) $$w_{n+1}=\frac{w_{n}+\lambda \left(\nabla_{w}f\left(w_{n}\right)\right)}{∥w_{n}+\lambda \left(\nabla_{w}f\left(w_{n}\right)\right)∥}\cdot \sqrt{P_{t}}.$$

As considered in Appendix A, the iteration (23) can converge to a KKT point of $\prod_{1}$ [43,44]. Hence based on (23), an optimal transmit beamforming policy can be proposed as Algorithm 2.

**Algorithm 2** Optimal beamforming policy
1:Initiate $w_{0}$ with constraint (16). Set step size $\lambda =\lambda_{int}$, and the minimum step size $\lambda_{\min}$. Obtain $\left\{\xi_{i}\right\},i\in R$.2:Set n=0, calculate $R_{n}=f\left(w_{n}\right)$ by (12).3:
**while** $\lambda >\lambda_{\min}$ **do**
4: Update $w_{n}^{\prime }$ according to (23) and calculate $R_{n}^{\prime }=f\left(w_{n}^{\prime }\right)$.5: If $R_{n}^{\prime }>R_{n}$, update $R_{n+1}=R_{n}^{\prime }$, $w_{n+1}=w_{n}^{\prime }$ and n←n+1. Otherwise, update $\lambda =\frac{1}{2}\lambda$.6:
**end while**
7:Output $w_{n}$, $R_{n}$.


Note that, the convergence of Algorithm 2 is guaranteed by the fact that $R_{n}$ increases or λ decreases with the increase of *n*, and $R_{n}$ is upper bounded by $\xi_{N_{r}}$ and λ is lower bounded by $\lambda_{\min}$. Meanwhile, considering multiple KKT points could exist for the non-concave problem, at least 10 samples are required to obtain the optimal beamforming vector w that achieves the maximum single-hop transmission distance.

With CSI known at the transmitters T, we use Algorithm 2 to obtain the numerical result of w. The computational complexity of Algorithm 2 depends on the number of iterations *I*, where the maximum number of iterations to reach convergence in our settings is I=20. In each iteration step, the algorithm calculates the gradient $\nabla_{w}f\left(w\right)$ to update the beamforming vector $w_{n+1}$ according to (23). The number of multiplications required in calculating $\nabla_{w}f\left(w\right)$ is $O\left(LN_{t}^{2}N_{r}^{2}\right)$, the number of additions required is similar to that of multiplications.

## 5. Simulation Results

In this section, the simulations are designed to show the effectiveness of the proposed scheme by comparing its performance with the above-mentioned conventional schemes via MATLAB.

We simulate a network with *N* nodes randomly distributed in a $M\times Mm^{2}$ square area. Let $P_{t}/\sigma^{2}=1$. For the reason of simplicity, we assume that the initial energy of each node is $E_{\mathrm{ini}}$, and each transmission will consume 1 unit energy in total during one time slot. The channel $h_{ij},\forall i\neq j$ follows independent and identically distributed (i.i.d.) Rayleigh fading, specifically, ${\left|h_{ij}\right|}^{2}=\phi_{ij}^{2}\cdot \gamma_{0}\cdot {\left(d_{i,j}/d_{0}\right)}^{-\alpha }$, where $\phi_{ij}$ follows the standard Rayleigh fading, $\gamma_{0}\cdot {\left(d_{i,j}/d_{0}\right)}^{-\alpha }$ is the path loss model, α is the path loss exponent, $d_{0}$ is a reference distance and $d_{i,j}$ denotes the distance between two nodes. Similar to [14,19,35,40], the exact simulation parameters are given in Table 1. The time is divided into sessions, and a source-to-destination pair generates in the network during each session. Each source has one packet to transmit to the destination.

The maximum ARQ retransmission attempts $N_{t}^{max}=1$, means that if the same node acts as forwarder or CH node more than two time slots, the packet will be dropped and the packet transmission will be deemed as failure. Moreover, in the following simulations, besides the conventional ExOR, AODV-ETX, and CCPT schemes, we introduce the ‘OCPT-FAR’ scheme as the baseline for performance comparison. In the ‘OCPT-FAR’ scheme, the transmit beamforming vector is always designed to align with $h_{i}$, where node *i* is the receiver with the farthest single-hop transmission distance, $i=\underset{i\in R}{\arg\max}\left\{\xi_{i}\right\}$. The results of the proposed OCPT scheme with optimized beamforming policy are labeled as the ‘OCPT-OPT’ scheme.

The performance metrics include the total number of packets arriving at destinations, packet arrival ratio, end-to-end transmission delay and transmission efficiency. We define the dead node as a node whose energy is depleted. The network lifetime is defined here as the time when the first dead node appears, and it can be measured with the number of sessions. The total number of packets arriving at destinations is tallied during the network lifetime. The packet arrival ratio indicates the ratio of the number of packets successfully arriving at destinations to the number of packets transmitted from the source nodes. Transmission efficiency is the number of packets successfully arriving at destinations divided the total time slots. The simulation results are averaged over node locations and channel realizations.

The first simulation is designed to show the end-to-end transmission delay and packet arrival ratio versus different number of nodes (*N*). Specially, the source $V_{s}$ and destination $V_{d}$ are fixed at left-down corner (0, 0) and right-up corner (100, 100) of the square area, respectively, and the remaining (N−2) nodes are randomly distributed in the area between $V_{s}$ and $V_{d}$. Set the number of packets to be transmitted from $V_{s}$ to $V_{d}$ as 2000, and the initial energy of each node is set as $E_{\mathrm{ini}}$ = 6000 energy units to guarantee the sufficient network lifetime.

**Figure 2:**
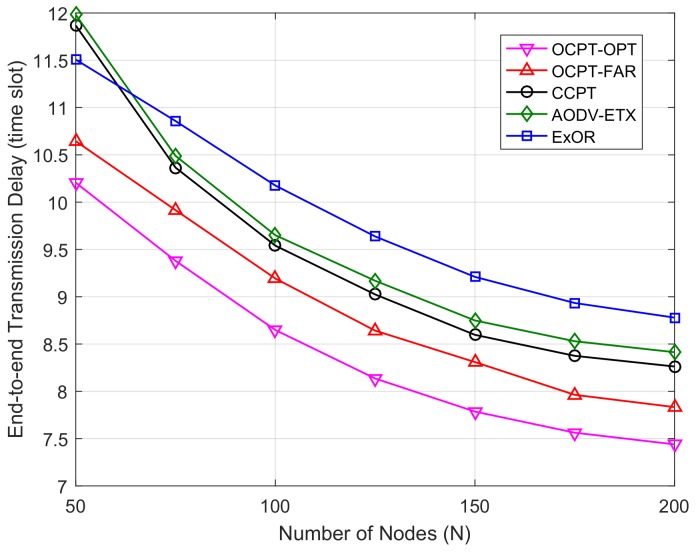
End-to-end transmission delay vs. number of nodes with different schemes under the fixed $V_{s}$ and $V_{d}$.

Figure 2 shows the end-to-end transmission delay versus the different number of nodes *N*. It can be seen from the figure that the curves of five schemes are decreasing as *N* increases, this is because when the number of nodes between $V_{s}$ and $V_{d}$ increases, the link between neighbors becomes more reliable, and less retransmission results in the lower transmission delay. Moreover, Figure 2 also shows that the end-to-end transmission delay with the proposed OCPT-OPT scheme is the lowest compared with the other schemes, which proves that the proposed scheme can effectively lower the transmission delay. The OCPT-OPT scheme outperforms the conventional ExOR scheme due to the gains of cooperative diversity. Meanwhile, the OCPT-OPT scheme has achieved a lower end-to-end transmission delay than optimal path based schemes (AODV-ETX, CCPT) because of the advantages of opportunistic routing. Moreover, OCPT-OPT scheme outperforms OCPT-FAR scheme in terms of transmission delay, which proves the effectiveness of the proposed optimal cooperative beamforming policy.

**Figure 3:**
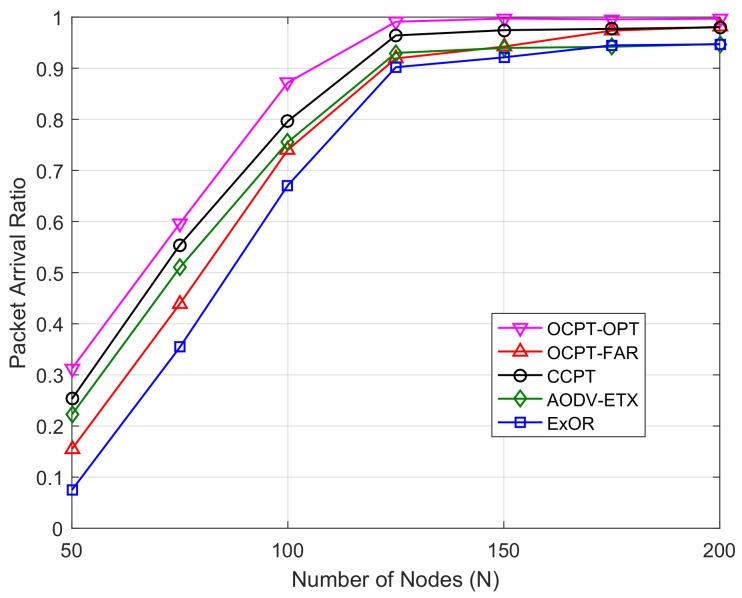
Packet arrival ratio vs. number of nodes with different schemes under the fixed $V_{s}$ and $V_{d}$.

Figure 3 shows the packet arrival ratio versus the different number of nodes *N*. We can observe from the figure that the packet arrival ratio increases with the increase of *N*, this is because a denser network improves the cooperative diversity and shortens the distance between neighbors, so that a higher end-to-end reliability can be achieved. Meanwhile, the packet arrival ratio of OCPT-FAR and ExOR is lower than that of CCPT and AODV-ETX when N≤150, and the gap is decreasing with the increase of *N*. This shows that in a sparse network, the packet transmission along with an elaborately-selected path is more reliable than the conventional opportunistic routing. The proposed OCPT-OPT has achieved the highest packet arrival ratio compared with the remaining four schemes, which shows that the optimized OCPT scheme has greatly improved the end-to-end transmission reliability of opportunistic routing.

The second simulation is performed to show the total number of packets arriving at destinations during the network lifetime versus different number of nodes (*N*). The source-destination pair randomly generates during each session. The initial energy of each node is set as $E_{0}=50$ energy units and the simulation terminates while the first dead node appears.

**Figure 4:**
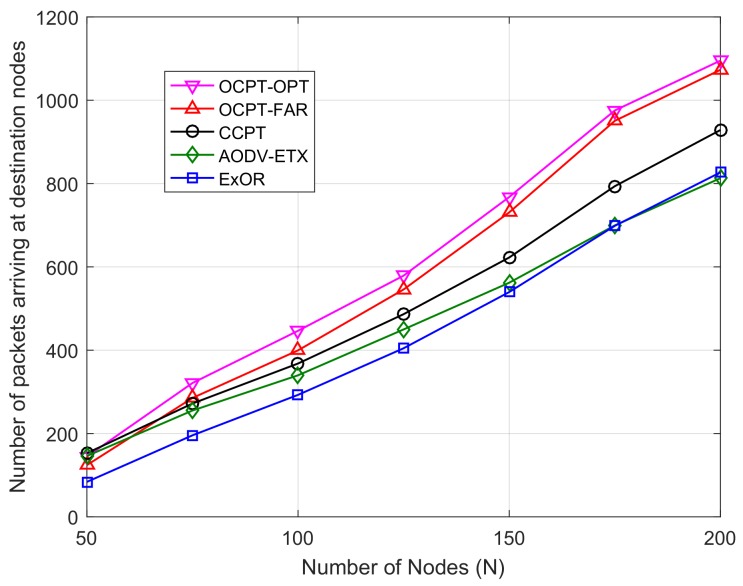
Number of packets successfully arriving at the destinations vs. number of nodes with different schemes under the random $V_{s}$ and $V_{d}$.

Figure 4 shows the performance with *N* ranging from 50 to 200. As shown in Figure 4, with the increase of *N*, the total number of packets arriving at destination nodes increases. This is because on the one hand, the increase of density of nodes can reduce the distance between neighbor nodes and improve the packet arrival ratio. On the other hand, the total energy of nodes increases as *N* increases, which prolongs the network lifetime. We can observe that when N=50, no significant gain is found between the performance of OCPT-OPT and CCPT (OCPT-FAR is even lower than CCPT and AODV-ETX in terms of the performance), this is because the packet arrival ratio of opportunistic routing in a sparse network is lower than that of optimal path based routing as shown in Figure 3. More importantly, no energy will be consumed in the network using CCPT and AODV-ETX once the initial path of AODV-ETX is not available, but the proposed OCPT-OPT and OCPT-FAR cannot pre-select the end-to-end route, and energy will be always consumed to transmit the data packet until the packet is received or dropped. Moreover, when N>50, it can be seen from Figure 4 that the performance of the proposed OCPT-OPT scheme outperforms that of other schemes, which proves that OCPT-OPT scheme can effectively improve the capacity of packet transmission for the network.

The third simulation is designed to show the effectiveness of the proposed OCPT scheme in terms of packet arrival ratio and transmission efficiency. To give a fair comparison, we fix the total number of packets to be transmitted from the source nodes as 2000. The initial energy $E_{0}$ of each node is set as 6000 energy units to guarantee the sufficient network lifetime. Similar to the second simulation, during each session, the source-destination pair randomly generates.

**Figure 5:**
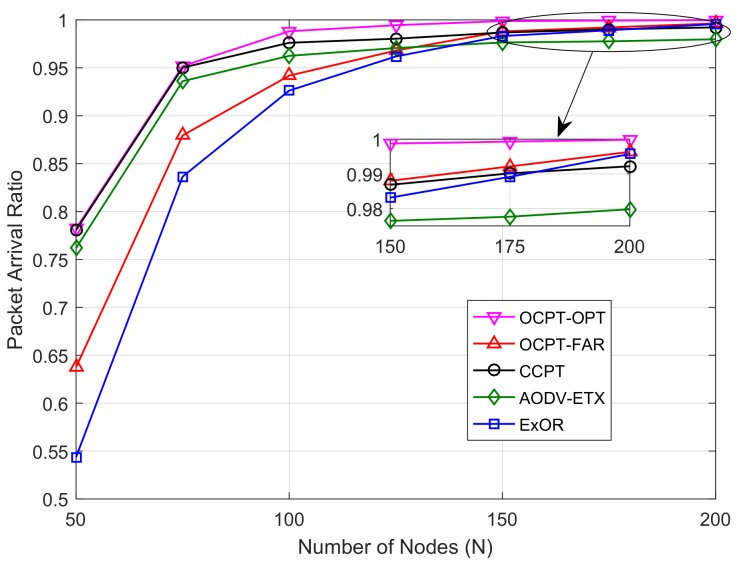
Packet arrival ratio vs. number of nodes with different schemes under the random $V_{s}$ and $V_{d}$.

Figure 5 shows the packet arrival ratio versus the number of nodes under the different schemes. Compared with the first simulation, the end-to-end transmission delay is obviously lower because of the randomly generated source-destination pair. Therefore, the packet arrival ratio in Figure 5 is higher than Figure 3. Moreover, similar to Figure 3, in Figure 5, the gap between the optimal path based schemes (AODV-ETX and CCPT) and opportunistic routing schemes (ExOR and OCPT) keeps decreasing. By contrast, when N=200, the packet arrival ratio of OCPT-FAR and ExOR is higher than that of CCPT and AODV-ETX. This shows that in a dense network, opportunistic routing schemes can achieve a better end-to-end transmission reliability than the optimal path based schemes. Furthermore, Figure 5 also shows the proposed OCPT-OPT scheme outperforms the remaining four schemes, which shows that optimal beamforming policy can largely improve the end-to-end transmission reliability whether in a sparse or dense network.

**Figure 6:**
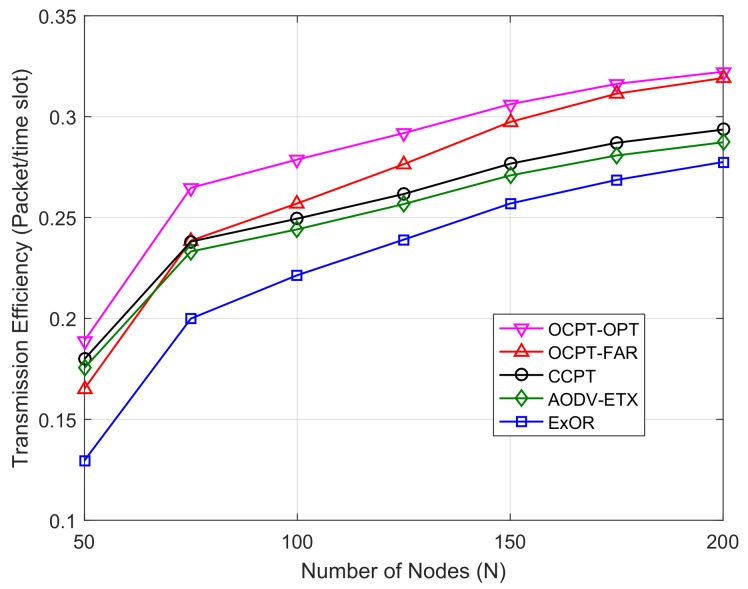
Transmission efficiency vs. number of nodes with different schemes under the random $V_{s}$ and $V_{d}$.

Figure 6 shows the transmission efficiency versus the number of nodes with different schemes. As shown in Figure 6, the transmission efficiency increases with the increase of *N*, which is because a denser network can improve the packet arrival ratio and further reduce the retransmission. Meanwhile, with the increase of *N*, the performance of opportunistic routing schemes (OCPT and ExOR) grows faster than the optimal path based schemes (AODV-ETX and CCPT), this is because the opportunistic routing can make better use of the neighbors for packet transmission. Moreover, similar to the result of Figure 5, it can be seen from Figure 6 that curve of the transmission efficiency with the proposed OCPT-OPT scheme is higher than that of other schemes, which demonstrates that the OCPT-OPT scheme can improve the successful packet transmission per time slot whether in a sparse or dense network.

## 6. Conclusions

In this paper, an OCPT scheme combining cooperative transmission and opportunistic routing is proposed to improve the end-to-end transmission performance in the wireless multi-hop networks. Based on the idea of opportunistic routing, CH is introduced as the organizer of each packet transmission, and multiple transmitters and receivers are further determined from the neighbor nodes of CH to form a cluster. Then, each intra-cluster cooperative packet transmission is formulated as a transmit beamforming optimization problem, and an iterative optimal beamforming policy is proposed to solve the problem and maximize the single-hop transmission distance. The simulation results show that the network with the proposed scheme can support a higher number of successful packet transmissions in comparison with other existing routing schemes. Furthermore, the proposed scheme can effectively shorten the end-to-end transmission delay, improve the packet arrival ratio and transmission efficiency.

It has been assumed in this paper that there is only one active communication session in the whole network. In case there are multiple active flows, the cooperative beamforming should address the interference that may occur at some relay nodes, for example with multi-channel operation [45], interference aware beamforming [46,47], non-orthogonal multiple access (NOMA), local scheduling [48], full-duplex operation [49] and etc. Future work will extend the proposed OCPT scheme to multiple-source multi-destination scenarios. To this end, the interference between different data flows needs to be carefully addressed.

## Figures and Tables

**Table 1:** Parameters for Simulation.

Parameter	Value	Parameter	Value
*M*	100 m	*Q*	4
*L*	512 bits	$N_{t}^{max}$	1
$d_{0}$	10 m	$\gamma_{0}$	18.5 dB
α	3	$N_{P}$	5
$\lambda_{int}$	1	$\lambda_{\min}$	0.001

**Figure A1:**
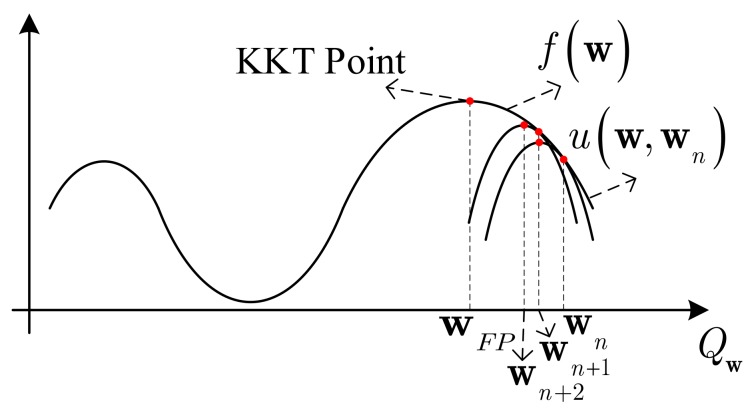
Convergency of the proposed iterations. Karush–Kuhn–Tucker (KKT).

## Appendix A. Convergency Analysis of (23)

According to solution (23) of problem $\prod_{2}$, the iteration is equivalent to a projected gradient update including the following two steps:

Step 1: Update $w_{n}$ in the feasible direction
  (A1) $${\tilde{w}}_{n+1}=w_{n}+\lambda \nabla f\left(w_{n}\right),$$
  λ is a positive step size.
Step 2: Project ${\tilde{w}}_{n+1}$ to the boundary set of 2-norm ball: $Q_{w}=\left\{w:{∥w∥}_{2}=\sqrt{P_{t}}\right\}$
  (A2) $$w_{n+1}=P_{Q_{w}}\left({\tilde{w}}_{n+1}\right),$$
  where $P_{Q_{w}}\left(\cdot \right)$ is the projection of the argument onto the feasible set $Q_{w}$.

Figure A1 shows the iterative process of (23). It can be noted that only when $w_{n+1}$ is the stationary point $w_{FP}$, i.e.,

(A3) $$w_{FP}=P_{Q_{w}}\left(w_{FP}+\lambda \nabla f\left(w_{FP}\right)\right),$$

the iteration will stop. The projected gradient update method can be used to search the local maximum of the constrained maximization problem $\prod_{1}$ in (17).

Using the convergence results for the projected gradient method in [43], it can now be shown that the iterations of (23) converge to a KKT point of (17).
